# Supplementary material for: Scalable training of artificial neural networks with adaptive sparse connectivity inspired by network science
Source: Nat Commun. 2018 Jun 19;9:2383. doi: 10.1038/s41467-018-04316-3 (PMC6008460; doi:10.1038/s41467-018-04316-3)
Supplement: Supplementary file 1 — Supplementary Information [file 41467_2018_4316_MOESM1_ESM.pdf]

Supplementary Information: Scalable Training of Artificial Neural Networks with Adaptive Sparse Connectivity inspired by Network Science

Mocanu et al.[*]

[*]Corresponding author: d.c.mocanu@tue.nl

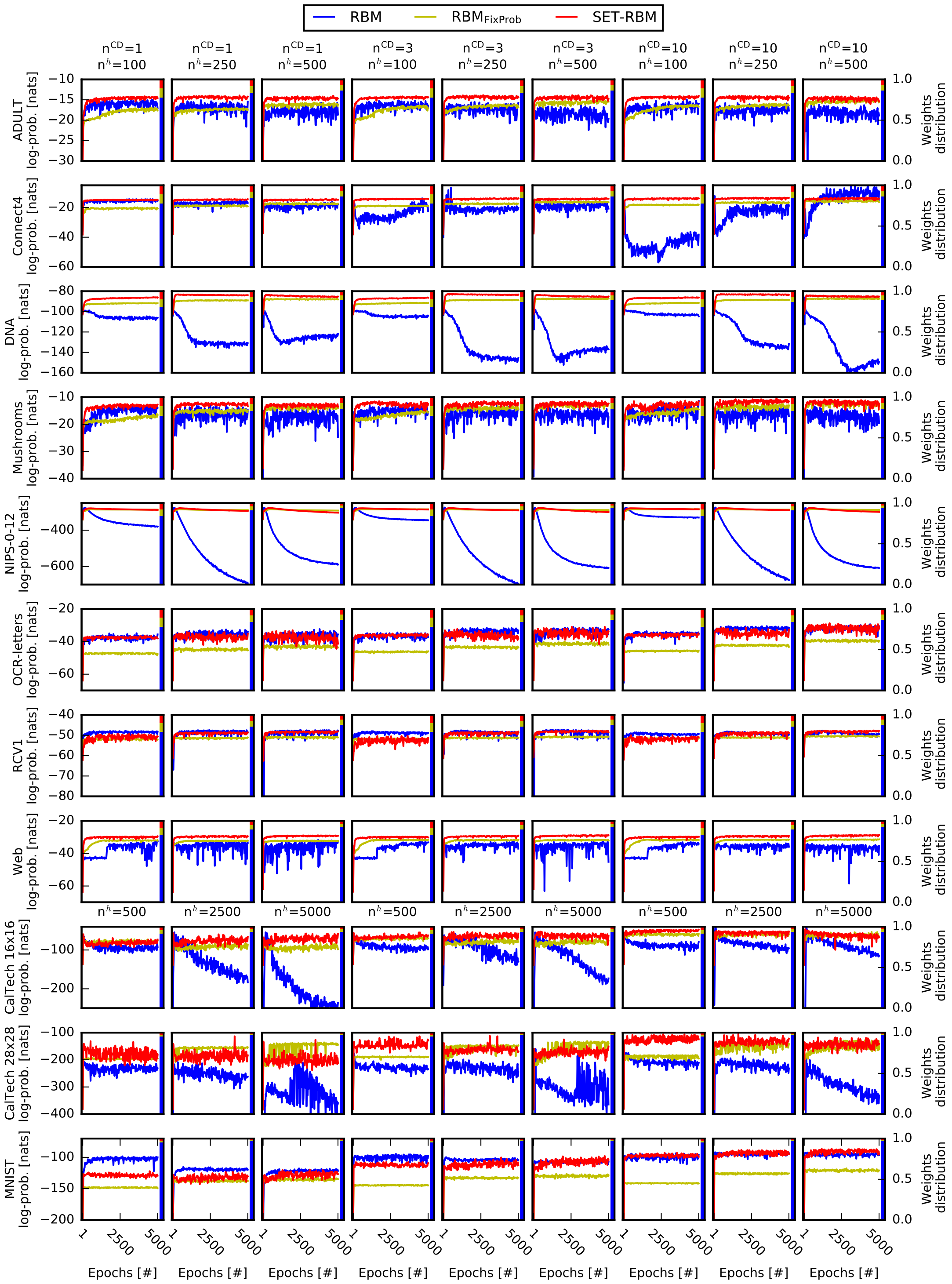

Supplementary Figure 1. Experiments with RBM variants using 11 benchmark datasets. For each model studied we have considered three cases for the number of Contrastive Divergence steps $n^{CD} = \{1, 3, 10\}$, and three cases for the number of hidden neurons (n^h). For the first 8 datasets (from top to bottom) we have used $n^h = \{100, 250, 500\}$, and for the last three datasets we have used $n^h = \{500, 2500, 5000\}$. The x-axes show the training epochs; the left y-axes show the average log-probabilities computed on the test data with Annealed Importance Sampling(AIS); and the right y-axes (the stacked bar on the right part of the plots) reflect the fraction given by the n^W of each model over the sum of the n^W of all three models. Overall, SET-RBM outperforms the other two models in most of the cases. Also, it is interesting to see that SET-RBM and $\text{RBM}_{\text{FixProb}}$ are much more stable and do not present the over-fitting problems of RBM.

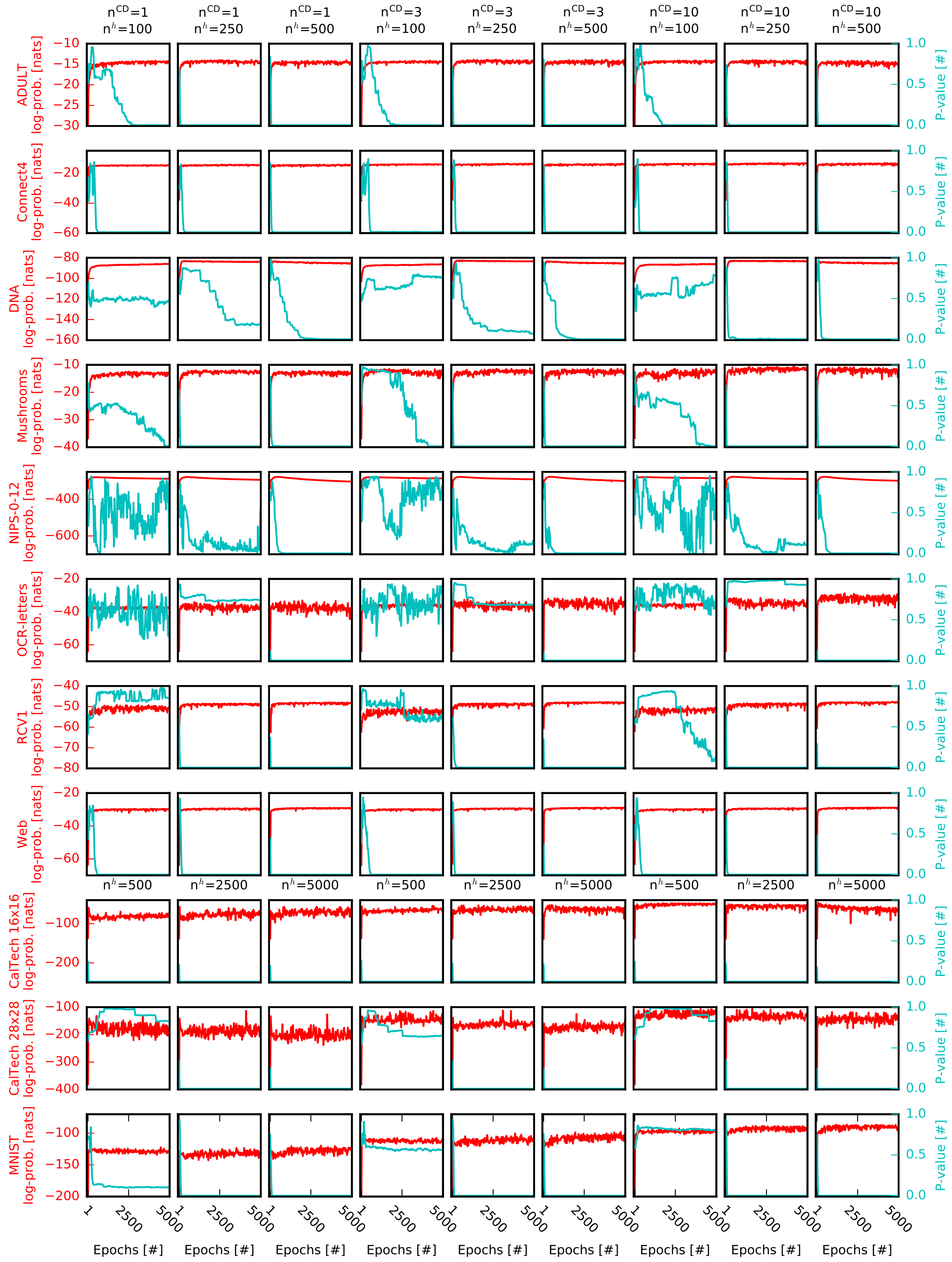

Supplementary Figure 2. SET-RBM evolution towards a scale-free topology. We have considered three cases for the number of Contrastive Divergence steps $n^{\mathrm{CD}} = \{1, 3, 10\}$, and three cases for the number of hidden neurons (n^h). For the first 8 datasets (from top to bottom) we have used $n^h = \{100, 250, 500\}$, and for the last three datasets we have used $n^h = \{500, 2500, 5000\}$. The x-axes show the training epochs; the left y-axes (red color) show the average log-probabilities computed for SET-RBMs on the test data with AIS; and the right y-axes (cyan color) show the p-values computed between the degree distribution of the hidden neurons in SET-RBM and a power-law distribution. We may observe that for models with a high enough number of hidden neurons, the SET-RBM topology always tends to become scale-free.
